# P019: Resistance evaluation of Escherichia coli strains isolated from urines in the urban environment in Benin

**DOI:** 10.1186/2047-2994-2-S1-P19

**Published:** 2013-06-20

**Authors:** L Guedezounme, M Chodaton, TA Ahoyo, H Bankole

**Affiliations:** 1 GBH / EPAC, University of Abomey-Calavi, Abomey, Benin

## Introduction

Antibiotic resistance represents a serious public health problem , particularly in resource limited rcountries. So, it is necessary to have an actualized knowledge about the resistance profile of these microorganisms.

## Objectives

To determine the resistance profile of *Escherichia coli* strains isolated from urines.

## Methods

This survey assessed 3678 samples of urines received at the national laboratory from 2009 to 2011. The tests used are urines cytobacteriologic exam and the disk diffusion method.

## Results

In total, 928 Enterobacteriaceae strains were isolated including 52.1% of *E. coli*, followed by *Klebsiella pneumoniae* (14%) and *Proteus mirabilis* (8.1%). *E. coli* occurred more frequently in women (75%). The antimicrobial susceptibility of *E. coli* shown that the highest rate of resistance was manifested against Amoxicillin (69%), then followed Amoxicillin + clavulanic Acid (51.1%), Tetracyclin (45.1%), Triméthoprim-sulfamethoxazole (42%), and nalidixic acid (35%), Ciprofloxacin (23.5%), Chloramphenicol (22.1%), Gentamicin (8.1%), and Ceftriaxon (5.1%). Multiresistance concerned 32% of tested strains.

## Conclusion

*E. coli* strains tested have developed more resistances against commonly prescribed antibiotics in clinical practice in Benin, which is not likely to facilitate the appropriate treatment of patients.

## Disclosure of interest

None declared

